# Fournier's Gangrene of the Penis following a Human Bite Wound

**DOI:** 10.1155/2018/9798607

**Published:** 2018-10-25

**Authors:** Tyler Overholt, Ali Hajiran, Cristiane Ueno, Stanley Zaslau

**Affiliations:** ^1^West Virginia University School of Medicine, USA; ^2^West Virginia University Department of Urology, USA; ^3^West Virginia University Department of Surgery, USA

## Abstract

Fournier's gangrene isolated to the penis is exceedingly rare. It is an urologic emergency that requires emergent parenteral antibiotics as well as aggressive irrigation and debridement. While human bite wounds can be overlooked as a serious cause of injury and infection, they can result in highly dangerous, polymicrobial infections in affected patients. Here, we report a case of penile Fournier's gangrene caused by a human bite wound managed with broad spectrum antibiotics, irrigation and debridement, penile reconstruction, and skin grafting with successful preservation of a normal penile structure and function.

## 1. Introduction

Fournier's gangrene is defined as necrotizing fasciitis involving the external genitalia, scrotum, or perineum, and involvement isolated to the penis is extremely rare. It is a surgical emergency in which rapid onset of management with broad spectrum intravenous antibiotics and surgical irrigation and debridement is necessary to prevent potentially fatal consequences. Human bite wounds, while often overlooked as a cause of a serious life threatening injury, can result in a fulminant, polymicrobial infection. We present a case of Fournier's gangrene isolated to the dorsal glans and shaft of the penis following a human bite wound.

## 2. Case Presentation

A 44-year old male with medical history of morbid obesity, diabetes mellitus, end stage renal disease, and osteomyelitis presented to our emergency department (ED) with the chief complaint of penile swelling. Nine days prior to presentation, the patient sustained an unintentional bite injury to the penis while receiving oral intercourse. Following the injury, he described worsening swelling, redness, penile discharge, pain, and inability to retract foreskin due to pain. The patient was initially treated for suspected balanitis with a seven-day course of an oral first generation cephalosporin, Keflex, and an oral anti-fungal, fluconazole, with plans for outpatient follow-up in the urology clinic. When the patient presented to the urology clinic the following week, he was found to have worsening tenderness and induration of his penis with phimosis and purulent drainage. An urgent computed tomography (CT) scan was performed showing subcutaneous emphysema involving the dorsal aspect of the penis concerning for a necrotizing soft tissue infection. The patient was subsequently taken to the operating room urgently for penile exploration and debridement.

**Figure 1:**
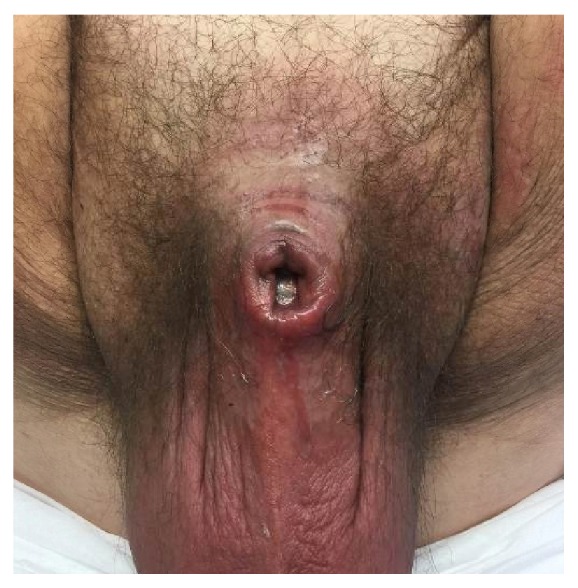
Initial examination revealing phimosis with purulent drainage.

**Figure 2:**
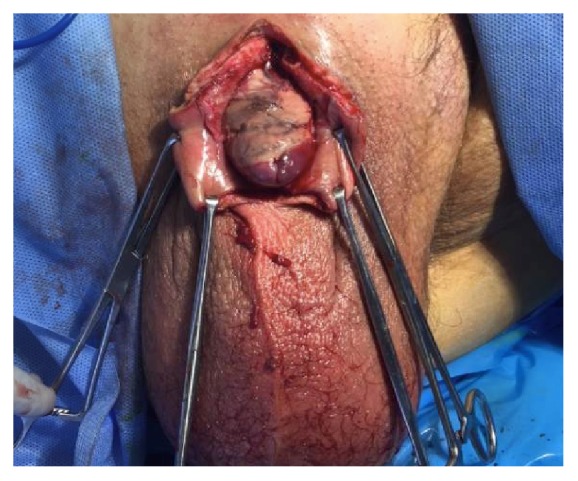
Dorsal and ventral slits with exposure of the glans revealing nonviable tissue on the dorsal aspect of the penis.

**Figure 3:**
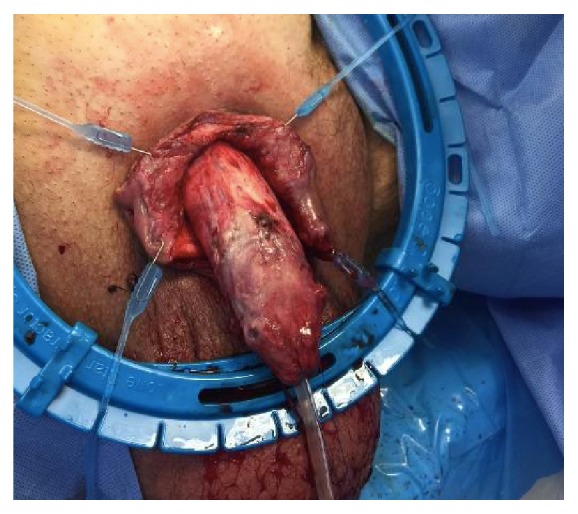
Remaining tissue appeared viable following degloving and debridement of the entire penis.

Examination under anesthesia demonstrated phimosis with purulent drainage from the phimotic ring as well as induration of the penile shaft (Figure 1). A dorsal midline incision was made through the foreskin to expose the glans of the penis and the penis was completely degloved down to the base. There appeared to be necrotic, nonviable tissue involving the dorsal aspect of the glans and shaft of the penis (Figure 2). All nonviable tissue was sharply debrided and the remaining tissue of the proximal shaft and ventral aspect of the penis appeared viable (Figure 3). The penis was irrigated using a PulsaVac^R^ and the edges of the foreskin were reapproximated with running 3-0 chromic suture. The penis was dressed with Xeroform^R^ gauze and Kerlix moistening in saline. Preliminary culture results obtained from the necrotic tissue collected during the surgery revealed a likely polymicrobial infection. Therefore, treatment with intravenous clindamycin, cefepime, and vancomycin was initiated.

**Figure 4:**
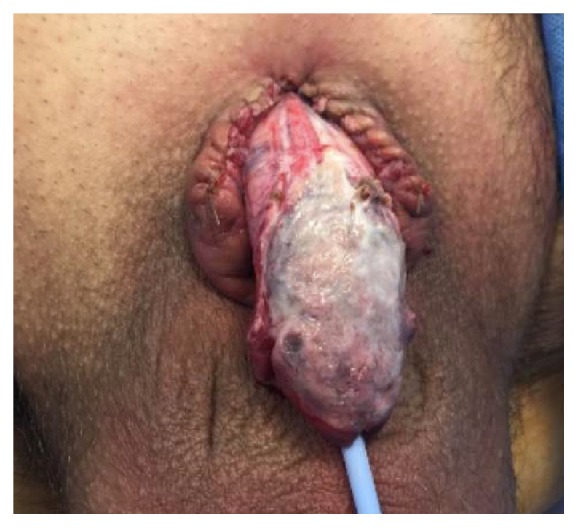
Necrotic tissue of the dorsal glans and shaft following initial irrigation and debridement.

**Figure 5:**
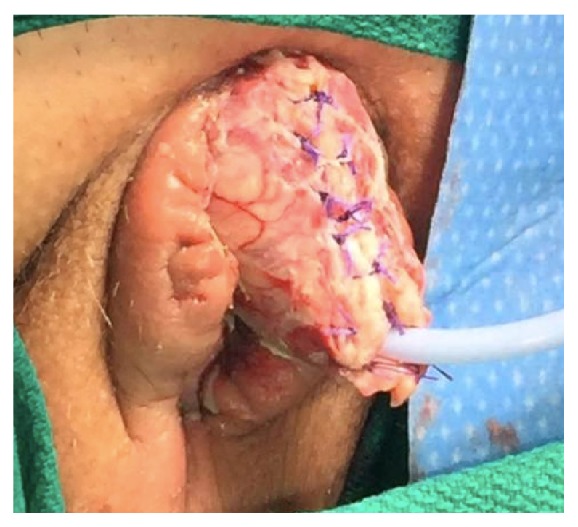
Remaining penile shaft after glansectomy and partial penectomy.

Two days following the initial debridement, the patient returned to the operating room (OR) for a repeat exam under anesthesia and further debridement. On initial exam, the dorsal aspect of the glans and shaft of the penis were found to be necrotic and nonviable (Figure 4). All nonviable tissue was then sharply excised from the dorsal aspect of the glans moving ventrally. As there was no visible, salvageable glandular tissue, we performed a complete glansectomy using sharp dissection. The necrotic tissue on the dorsal aspect of the penile shaft was shaved until we encountered viable, bleeding tissue from the corpora of the mid-shaft. The distal urethra was sharply resected during removal of the glans of the penis. The distal urethral remnant was then spatulated in the epithelial edges of the new widely patient urethral meatus and the edges were reapproximated using interrupted 3-0 Vicryl suture. The corpora of the penile shaft was then reapproximated in a tubularized fashion using interrupted 2-0 Vicryl suture through the tunica albuginea (Figure 5). Once the shaft was reapproximated and hemostasis had been ensured, PulsaVac^R^ was used to irrigate the wound with 3 L of sterile normal saline and the penile shaft was dressed with Kerlix moistened in saline. Plastic surgery was consulted for intraoperative evaluation with possible grafting and/or other types of wound coverage with plans for returning to the OR.

**Figure 6:**
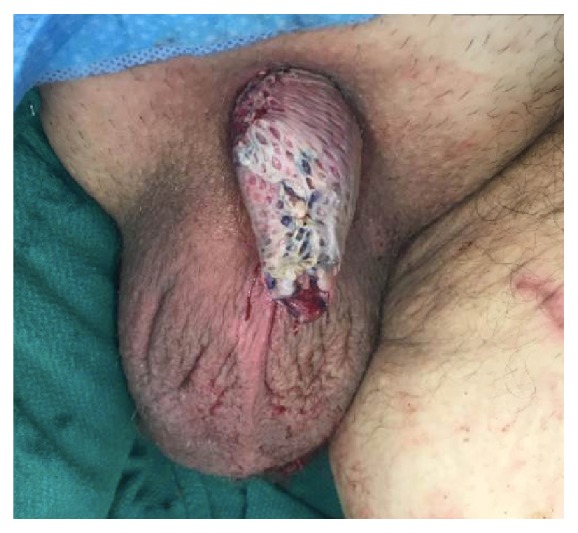
Dorsal shaft following placement of split thickness skin graft.

**Figure 7:**
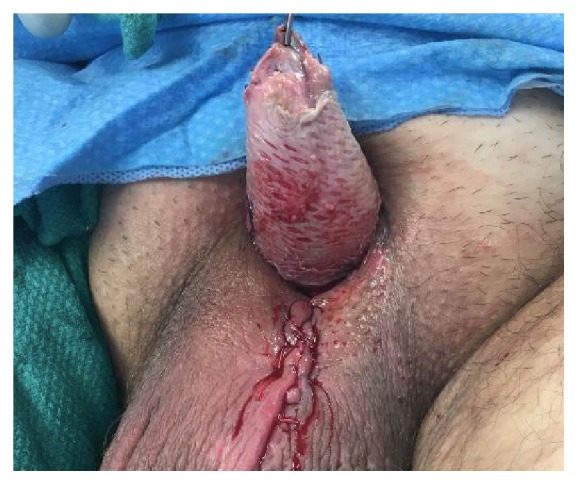
Ventral view of shaft following placement of split thickness skin graft.

Five days later the patient returned to the OR for a final surgery, which included wound coverage. On initial exam, the patient had 6 cm of residual penile shaft. There was fibrinous material on the left lateral aspect of the dorsal shaft and around the base of the penis. All remaining nonviable tissue was debrided. We then used the PulsaVac^R^ to irrigate with 3 L of normal saline mixed with 160 mg of gentamicin. The soft tissue defect around the base of the penis was closed using 4-0 Monocryl interrupted vertical mattress suture. Anchoring sutures were placed at the dorsal base of the penis between Buck's fascia and the dermis of the skin using interrupted 3-0 Vicryl. Length and width of the shaft following closure at the based of the penis measured 7 cm and 9 cm, respectively, resulting in about 1 cm of penile shaft length gained by adding these sutures. We then turned our attention towards the wound coverage. We opted for split thickness skin grafting given the wound bed viable aspect. A rectangular area of the left anterior thigh was chosen as a donor site for skin harvest. Using a dermatome, a split thickness skin graft measuring 0.1 – 0.2 mm in depth, 7 cm in length, and 9 cm in width was harvested. The graft was wrapped around the shaft of the penis and secured into place using anchoring interrupted 4-0 chromic suture (Figures 6 and 7). Once the graft was secured on the shaft of the penis, a new 16 Fr Foley was placed, followed by application of a MepiTEL AG^R^ (Molnlyche) dressing that was wrapped around the graft to protect from adhering to negative pressure therapy device (Wound V.A.C.^R^) foam, and the shaft was covered using 2 pieces of black foam and wound V.A.C.^R^ drape.

The patient continued to do well postoperatively. Intraoperative cultures ultimately grew* Escherichia coli, Enterococcus avium*, and* Gamella morbillorum*. He remained on broad spectrum IV antibiotics throughout his hospitalization for a total of 13 days. He was discharged home with 2 weeks of Amoxicillin-clavulanate and Clindamycin. He returned to urology and plastic surgery clinic three weeks later for follow-up where he demonstrated overall clinical improvement. His penile wounds continued to heal and his Foley catheter was removed.

## 3. Discussion

Fournier's gangrene is a fulminant necrotizing fasciitis of the external genitalia, scrotum, or perineal area. Men are affected exceedingly more than women, and the majority of patients suffer from multiple health comorbidities including obesity, diabetes, and immunodeficiency [1]. Clinical findings include fever, pain and swelling, erythema or dark discoloration, discharge from the wound, induration, and crepitus of the affected area [2]. A key component in accurate diagnosis is imaging with CT being the study of choice. Important findings include fascial thickness and subcutaneous air [2]. The pathogenesis is usually bacterial and the most common organisms identified include* Escherichia coli, Klebsiella pneumoniae, Staphylococcus aureus, Bacteroides fragilis, Streptococcus *species*, and Clostridium *species [1, 2]. Over 50 species of bacteria have been identified in the oral flora as well, including* Streptococcus species, staph aureus, Eikenella corrodens, and Fusobacterium nucleatum* and thus human bite wounds can result in polymicrobial infections including fulminant necrotizing fasciitis that can require aggressive management [3, 4]. Commonly used antibiotic regimens for Fournier's gangrene include a second- or third-generation cephalosporin, fluoroquinolones or gentamicin, and clindamycin [1, 2]. Based on the organisms most commonly identified in both bite wounds and in Fournier's gangrene, we elected for antibiotic coverage with vancomycin, cefepime, and clindamycin for Gram positive, Gram negative, and anaerobic coverage. Length of antibiotic duration varies widely as there are no clear guidelines on management. One study compared outcomes in patients who received antibiotics for a scheduled number of days versus patients who stopped receiving antibiotics following surgical debridement and normalization of clinical indicators of infection and found that there was no significant difference in recurrence of infection [5].

Literature review revealed 14 total reported cases of isolated penile Fournier's gangrene and the first reported case of penile Fournier's gangrene caused by a bite wound was in 1976 [6]. The clinical severity in reported cases varies wildly. Cases of monomicrobial* Eikenella corrodens *infection from a human bite have been found to cause a locally destructive lesion that was managed with local irrigation and broad spectrum antibiotics [6]. Other cases caused by a polymicrobial infection required emergent irrigation and debridement distal to the affected area, broad spectrum IV antibiotics, and skin grafting [7, 8]. In this case, we demonstrated a rare incidence of penile Fournier's gangrene managed with broad spectrum IV antibiotics, irrigation and debridement, penile reconstruction, and skin grafting to aggressively control the infection while attempting to preserve both sexual and urinary function.
